# Current Perspectives on 2D and 3D Cell Culture Models in Cancer Research: Molecular Determinants of Tumor Biology and Therapeutic Response

**DOI:** 10.3390/cimb48090914

**Published:** 2026-09-06

**Authors:** Selma Yıldırım, Juan Gómez-Salgado, Luis El Khoury-Moreno, Özkan Özden

**Affiliations:** 1 Department of Biotechnology, Kafkas University, 36000 Kars, Türkiye; sselmayyildirim@gmail.com; 2 Department of Medicine and Public Health, Faculty of Labour Sciences, University of Huelva, 21007 Huelva, Spain; 3 Safety and Health Postgraduate Programme, Universidad Espíritu Santo, Guayaquil 092301, Ecuador; 4 Department of Stomatology, Faculty of Odontology, University of Seville, 41009 Seville, Spain; lelkhoury@us.es; 5 Department of Bioengineering, Kafkas University, 36000 Kars, Türkiye

**Keywords:** cancer, 2D cell culture, 3D cell culture, tumor microenvironment, extracellular matrix

## Abstract

Cancer remains a major global health challenge, requiring innovative diagnostic and therapeutic strategies. Cell culture models are essential tools in cancer research. While traditional two-dimensional (2D) cultures are widely used because of their accessibility and simplicity, they inadequately represent the tumor microenvironment. This review provides a molecular perspective on the biological differences between 2D and 3D cancer models, emphasizing extracellular matrix signaling, mechanotransduction, metabolic adaptation, tumor heterogeneity, and therapeutic resistance rather than a general comparison of culture systems. It discusses the limitations of 2D models and highlights the advantages of 3D systems, including spheroids and organoids, for more accurately recapitulating the tumor microenvironment and improving the predictive value of anticancer drug testing. This narrative review synthesizes current evidence on the roles of 2D and 3D cell culture methods in oncology. The literature was searched in PubMed, Google Scholar, and Web of Science from 2015 to 2026 using keywords including “2D cell culture,” “3D cell culture,” “spheroids,” “organoids,” and “cancer.” Studies were eligible for inclusion if they addressed cancer research using 2D or 3D cell culture models and provided relevant experimental, preclinical, or translational evidence, particularly regarding tumor microenvironment, extracellular matrix signaling, cellular interactions, therapeutic response, or molecular mechanisms. Studies unrelated to cancer or 2D/3D culture models, duplicate records, and publications lacking relevant primary data were excluded. Study selection was performed by screening titles and abstracts followed by full-text assessment according to the predefined eligibility criteria. Seminal studies were also included when considered relevant to the conceptual framework of the review. Findings were synthesized narratively without quantitative analysis. 2D cultures limit cell–cell and cell–extracellular matrix interactions, reducing the applicability of findings to in vivo conditions. In contrast, 3D cultures better reproduce oxygen and nutrient gradients and cellular interactions, providing a more realistic representation of the tumor microenvironment. Recent advances in 3D systems have improved predictive accuracy for drug screening and personalized medicine. Both 2D and 3D culture methods possess unique strengths and limitations. Used in complementary ways, they can improve the translation of in vitro findings to in vivo and clinical applications, accelerating progress in cancer research and therapeutic development.

## 1. Introduction

Cancer remains one of the most significant global health problems threatening human health due to its high mortality rates [1]. Cancer is a multifactorial group of diseases in which abnormal cells, normally eliminated by the immune system, escape immune surveillance through complex mechanisms, leading to uncontrolled proliferation, tumor formation, and disruption of tissue homeostasis [2]. Cancer cells achieve immune evasion by creating an immunosuppressive microenvironment and targeting key signaling pathways that regulate immune responses [3,4]. However, the metastatic potential of the disease makes clinical control more difficult. Primary tumor cells invade surrounding tissues and form lesions in other parts of the body, which increases malignancy and complicates disease management [2,5]. In this context, the complexity of cancer morphology, understanding the tumor microenvironment, and accurately evaluating therapeutic responses have driven researchers to develop more reliable and predictive experimental approaches. Accordingly, there is a need to develop experimental models that can more realistically reflect cancer biology.

Since their discovery, cell culture methods have been indispensable tools for researchers. Cell culture is an in vitro technique that plays a central role in modern oncology studies, enabling the analysis of complex processes in cancer biology and the development of new therapeutic and diagnostic approaches [6]. The selection of a suitable cell culture method in cancer studies allows for a more detailed understanding of the molecular and cellular processes underlying tumor biology [7]. Two-dimensional (2D) cell culture techniques, which enable cells to grow under laboratory conditions and enable the analysis of basic biological processes, have been widely used in cancer research for many years [8]. In this method, cells adhere to the surface as a monolayer within a liquid culture medium to maintain viability [7]. However, this condition significantly limits cell–cell and cell–extracellular matrix (ECM) interactions. Due to these limited interactions, cells cannot adequately mimic the tumor microenvironment and, therefore, may not exhibit drug responses similar to in vivo conditions [9]. Accurately and realistically modeling the tumor microenvironment remains one of the main challenges in cancer research. In this regard, three-dimensional (3D) cell culture models provide more effective and physiologically relevant experimental systems by better reflecting the structural and functional properties of in vivo tissues [10].

This narrative review synthesizes current evidence on the roles, biological relevance, methodological characteristics, limitations, and translational applications of 2D and 3D cell culture models in cancer research. The literature was searched in PubMed, Google Scholar, and Web of Science for studies published between 2015 and 2026 using keywords including “2D cell culture,” “3D cell culture,” “spheroids,” “organoids,” and “cancer.” Studies were eligible if they addressed cancer research using 2D or 3D cell culture models and provided relevant experimental, preclinical, or translational evidence, particularly regarding the tumor microenvironment, extracellular matrix signaling, cellular interactions, therapeutic responses, or underlying molecular mechanisms. Studies unrelated to cancer or 2D/3D culture models, duplicate records, and publications lacking relevant primary evidence were excluded. Study selection involved screening titles and abstracts followed by full-text assessment according to predefined eligibility criteria. Seminal studies were also included when considered relevant to the conceptual framework of the review. The evidence was synthesized narratively without quantitative analysis. Particular emphasis was placed on the methodological differences between 2D and 3D systems, their ability to reproduce key aspects of tumor biology, and their influence on cancer cell behavior and drug responses. The review also examines recent advances in 3D culture technologies, including spheroids and organoids, and their potential to improve the biological relevance and translational accuracy of cancer models. Given that no single culture system can fully recapitulate the complexity of tumor biology, the selection of an appropriate model should be guided by the specific research question and experimental objectives, while considering the inherent strengths and limitations of each approach.

### 1.1. Development of Cell Culture Systems in Cancer Research

Cell culture methods allow cells obtained from tissues to be grown under in vitro conditions where environmental parameters such as temperature, pH level, and growth factors are controlled, thereby reducing variability and enabling reproducible results [11]. The foundations of cell biology were laid in 1665 when Robert Hooke first observed and described cells [12]. Over the following centuries, advances in microscopy further contributed to the development of cell-related studies, and over time, cell culture methods evolved and became an essential part of biological research.

In 1910, Ross Harrison developed the first cell culture technique, which allowed the direct observation of living cells. In this method, tissue fragments were placed in a partially coagulated plasma or lymph medium on a glass surface, inverted, and observed under a microscope [13]. This method showed that cells could be sustained outside the organism when placed under carefully controlled conditions; however, it also revealed the need for further development of these techniques.

In 1912, Carrel and colleagues succeeded in maintaining cells obtained from tissue samples alive for a short period under in vitro conditions [14]. This advancement paved the way for the use of cell culture as an experimental method. One of the best-known examples of cell culture is the first immortalized human cell line, HeLa cells. These cells were isolated from cervical cancer tissue taken from Henrietta Lacks in 1951 and have since been widely used in many areas, including cancer research, drug development, and molecular biology [15].

These developments enabled the widespread use of cell culture, particularly in fields such as oncology and cancer biology. However, the establishment of cell culture as a reproducible and reliable experimental method has only been possible through technical advances such as the development of defined media, the inclusion of controlled environmental conditions, and the standardization of culture techniques [14].

### 1.2. Advantages and Limitations of 2D and 3D Cell Culture Models

Modeling tumor pathobiology is highly challenging because of the complex nature of the disease. Cancer cells exist within a heterogeneous and dynamic structure composed of stromal cells, immune cells, and the ECM [16]. Variations in oxygen and nutrient gradients, signals that drive phenotypic plasticity, and systemic factors further complicate the diverse cellular interactions within the tumor microenvironment. This complexity makes it difficult to fully replicate tumor structure using experimental models [17].

Two-dimensional (2D) cell culture systems remain fundamental experimental platforms in modern biomedical research due to their low cost, operational simplicity, and high reproducibility [18]. Cells growing as a homogeneous monolayer facilitate the easy integration of microscopic imaging, flow cytometry, and other molecular analyses into high-throughput screening (HTS) protocols [19]. This scalable structure also offers a significant advantage in standardizing functional genomics studies requiring high efficiency, such as CRISPR-Cas9 and RNA interference (RNAi). RPMI-1640 medium, which is widely used in cell culture, was originally developed for lymphoid cells but is now capable of meeting the metabolic requirements of a large number of adherent cell lines [20]. Whilst fetal bovine serum (FBS) added to the medium provides the growth factors necessary for cell proliferation, it has been shown that the use of human serum (HS) can increase the cells’ invasive capacity more markedly than FBS [21].

To mimic the ECM and enhance cellular adhesion, culture surfaces can be coated with ECM proteins, such as Type IV collagen, or modified with semi-synthetic biomaterials, such as gelatin methacryloyl (GelMA), which contains Arg-Gly-Asp (RGD) sequences [22]. However, one of the most significant molecular limitations of 2D culture systems is that cells are exposed to approximately 18.6 per cent atmospheric oxygen under standard culture conditions, leading to hyperoxic stress [23]. These supraphysiological oxygen levels lead to the accumulation of reactive oxygen species (ROS) within the cell, thereby increasing oxidative stress and threatening DNA integrity [22,24]. Furthermore, Mycoplasma infections, a common source of contamination, can impair cellular base excision repair mechanisms [25]. In contrast, unlike thick and opaque 3D tissues, the absence of light penetration issues in 2D monolayer cultures enables high-resolution microscopic imaging [18].

Genetic drift arising in long-term cultures can disrupt the genomic stability of cell lines, leading to variations in phenotypic characteristics between experiments [26]. Furthermore, as 2D models cannot replicate the oxygen and nutrient gradients found within tissue, the IC50 values determined in drug efficacy studies are often found to be lower than those observed in vivo [18]. The absence of cell polarization and nutrient gradients also prevents the complete replication of natural tissue physiology at the molecular level [27]. Although uniform access to oxygen for cells may reduce variation in large-scale omics datasets by making metabolic profiles more predictable, in terms of transcriptomic diversity and physiological realism, 2D culture systems fall short of the biological depth offered by 3D models [19].

**Figure 1:**
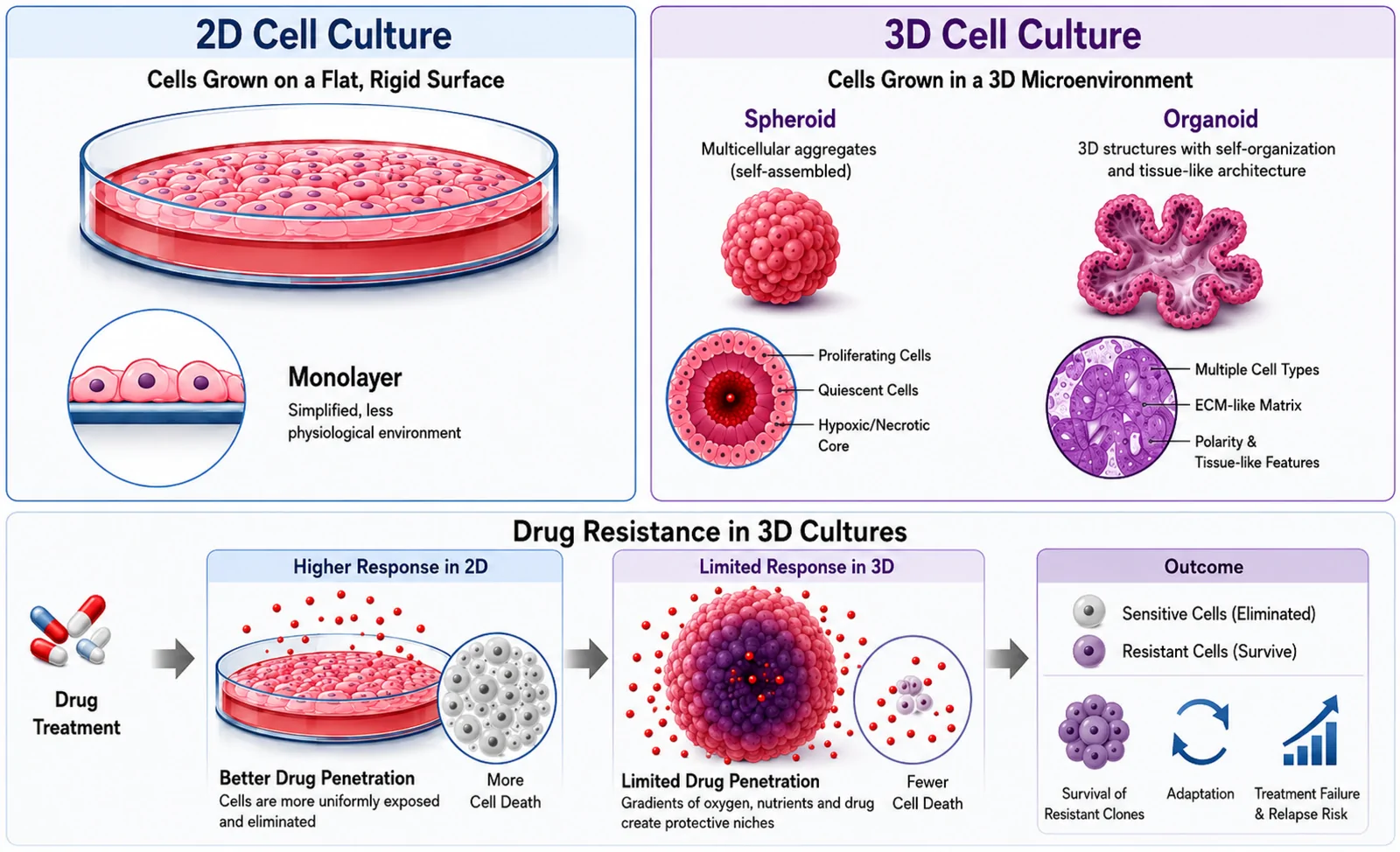
Molecular and functional differences between 2D and 3D cell culture models. This figure was generated using ChatGPT (OpenAI; GPT-5.6 Luna) through iterative, content-specific prompts describing and comparing 2D monolayer and 3D cell culture models with respect to cellular architecture and organization, cell–cell and cell–matrix interactions, extracellular matrix (ECM) composition, spatial organization, oxygen and nutrient gradients, drug penetration and distribution, cellular heterogeneity, and mechanisms associated with drug resistance. The generated output was subsequently reviewed and edited by the authors to improve its scientific accuracy, molecular and biological detail, visual clarity, consistency with the literature, and resolution. The authors take full responsibility for the scientific content, interpretation, and final version of the figure.

Taken together, these limitations restrict the ability of 2D cultures to reproduce the complexity of the tumor microenvironment [10] (Figure 1). Although animal models remain important for reproducing tumor biology under in vivo-like conditions, species-specific physiological and molecular differences continue to limit their translation [28]. These limitations have increased interest in 3D cell culture models in cancer research. Unlike traditional 2D systems, 3D culture techniques can mimic the tumor microenvironment more realistically, and the cellular responses observed towards drug treatments are known to be closer to in vivo outcomes [6,8,17]. The effectiveness of 3D cell culture models is closely associated with more advanced cell–ECM interactions [29].

Beyond serving as a structural scaffold in culture systems, the ECM is a dynamic three-dimensional network that regulates numerous biological processes in normal tissues [30]. It plays a central role in regulating essential biological processes such as cell adhesion, proliferation, differentiation, and migration. At the same time, it controls cell–cell interactions and microenvironmental signaling [31]. Under physiological conditions, the synthesis and remodeling of the ECM maintain tissue homeostasis, whereas disruption of this balance contributes to the development of many pathological conditions, including cancer [29,32].

In cancer biology, ECM is considered not only a passive structural component but also an active element of the tumor microenvironment. This highlights that tumor progression is influenced not only by genetic alterations but also by microenvironmental factors. The desmoplastic response, frequently observed in solid tumors, is characterized by collagen accumulation and increased tissue stiffness. These structural changes activate mechanotransduction pathways, promoting invasion, cell proliferation, and epithelial–mesenchymal transition (EMT). Therefore, abnormalities in the ECM play a complementary and regulatory role in tumor progression [33].

ECM components, which are key determinants of tumor cell behavior, are not only structural elements but are also considered prognostic and diagnostic biomarkers alongside potential therapeutic targets [34]. However, conventional 2D cell culture models, which cannot adequately represent the complex biochemical and biophysical properties of the ECM, limit the realistic study of the tumor microenvironment [6,10]. Considering all these findings, it can be stated that cell–ECM interactions in the culture environment are of great importance for obtaining reliable results. Therefore, 3D cell culture systems, in which cells are typically cultured in ECM-like hydrogel environments, provide biologically more relevant and translationally valuable in vitro models for cancer research [35] (Figure 1).

#### 1.2.1. Molecular Basis of Cell–Extracellular Matrix Interactions and Mechanotransduction

ECM is not only a structural component of the tumor microenvironment but also a key signaling platform that regulates cell behavior [36]. In particular, the accumulation of collagen and matrix stiffening that occurs during tumor development causes cells to perceive mechanical stimuli and convert them into biochemical signals [37]. In this process, integrins act as key mechanosensors and, upon binding to ECM components, trigger the formation of focal adhesion complexes [36,38]. This is followed by the activation of focal adhesion kinase (FAK) and Src family kinases, which in turn activate pathways such as PI3K/Akt and MAPK/ERK that regulate cell survival, proliferation, and metabolism [39]. In particular, the sustained activation of the PI3K/Akt signaling pathway contributes to the suppression of apoptosis and makes tumor cells more resistant to treatment, whilst the MAPK/ERK cascade supports cell cycle progression and proliferation [37,38].

**Figure 2:**
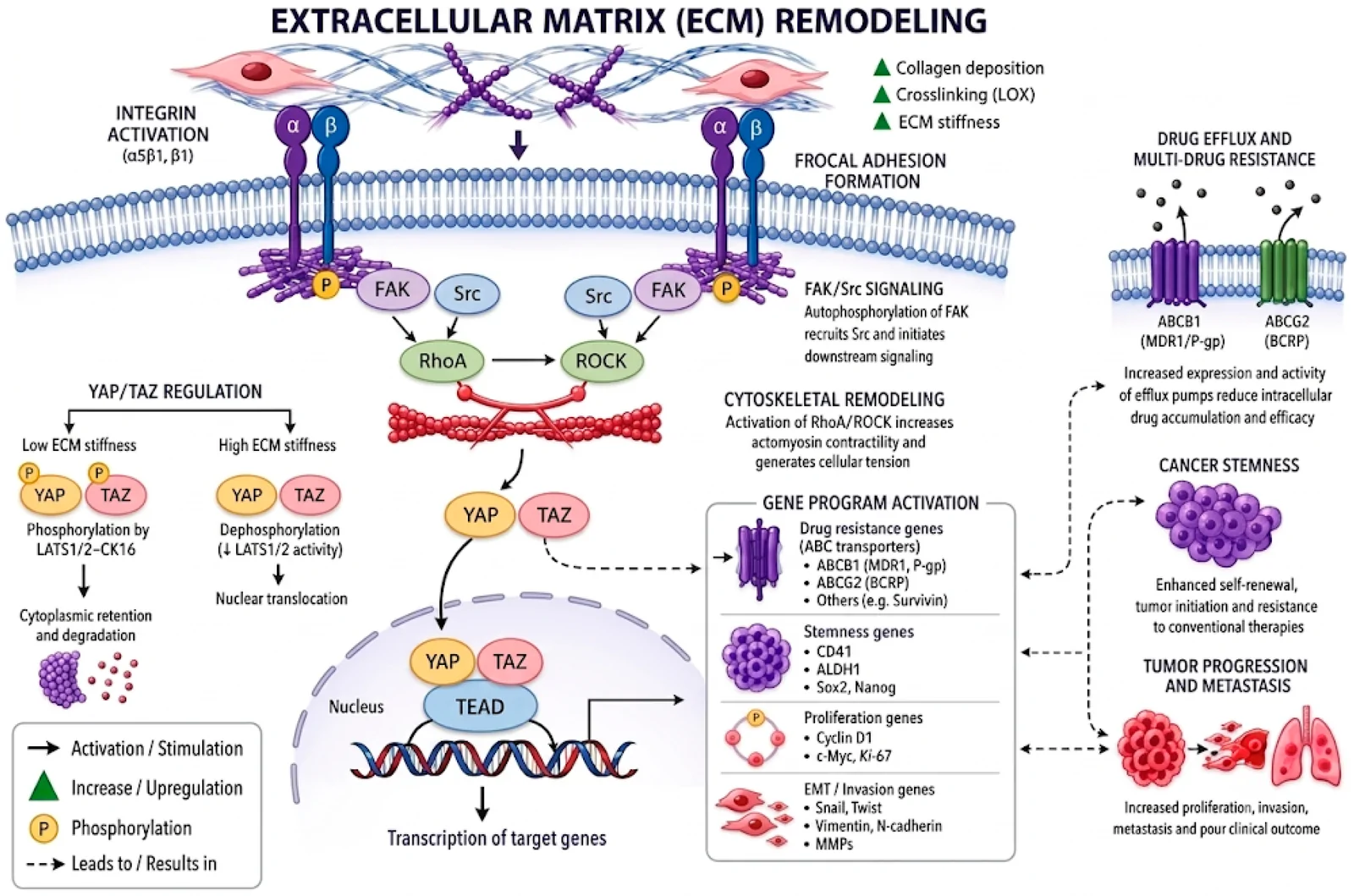
Increased extracellular matrix stiffness activates mechanotransduction pathways that promote tumor progression, metastasis, cancer stemness, and therapeutic resistance. This figure was generated using ChatGPT (OpenAI; GPT-5.6 Luna) through iterative, content-specific prompts specifying the molecular mechanisms linking extracellular matrix (ECM) remodeling and increased matrix stiffness to integrin activation, focal adhesion kinase (FAK)/Src signaling, RhoA/ROCK-mediated cytoskeletal remodeling, YAP/TAZ regulation, nuclear YAP/TAZ–TEAD interaction, and transcriptional activation of genes associated with drug resistance (ABCB1 and ABCG2), cancer stemness, proliferation, epithelial–mesenchymal transition (EMT), invasion, and metastasis. The prompts also specified the inclusion of ECM collagen deposition and LOX-mediated cross-linking, focal adhesion formation, actomyosin contractility, differential YAP/TAZ phosphorylation under low- and high-stiffness conditions, and the resulting effects on tumor progression and therapeutic response. The generated output was subsequently reviewed and edited by the authors to improve its scientific accuracy, molecular and mechanistic detail, visual clarity, resolution, and consistency with the literature. The authors take full responsibility for the scientific content, interpretation, and final version of the figure.

Another key component of mechanotransduction is the RhoA/ROCK signaling pathway (Figure 2), which regulates the organization of the cytoskeleton [36,40]. Matrix stiffness initiates the process of mechanotransduction by stimulating integrin receptor clustering and the focal adhesion kinase (FAK) signaling pathway in cancer cells within the tumor microenvironment [41]. This physical signal generates significant mechanical tension in the cytoskeleton by increasing actomyosin contraction via RhoA, a small GTPase, and its effector ROCK (Rho-associated protein kinase) [39]. Increased mechanical tension suppresses the Hippo signaling pathway, particularly the LATS1/2 cascade, which normally phosphorylates YAP/TAZ proteins and retains them in the cytoplasm [38]. Under these conditions, tension-dependent phosphatase activity also promotes YAP/TAZ dephosphorylation, facilitating their release from cytoplasmic 14-3-3 binding proteins and subsequent nuclear translocation [38]. In the nucleus, YAP/TAZ forms a robust complex with TEAD (TEA-domain) transcription factors, which regulates the expression of numerous genes associated with cell proliferation, invasion, angiogenesis, and EMT [37]. Importantly, YAP/TAZ–TEAD signaling also enhances the cancer stem cell phenotype and its self-renewal capacity by inducing the expression of master stem cell markers such as SOX2, OCT4 (POU5F1), and NANOG [42]. Concurrently, the YAP/TAZ–TEAD interaction upregulates the expression of ATP-binding cassette (ABC) drug efflux transporters, including ABCB1 (P-glycoprotein) and ABCG2 (BCRP), which actively pump chemotherapeutic drugs out of cancer cells and thereby contribute to chemoresistance [41]. Consequently, the integrin–FAK/Src–RhoA/ROCK–YAP/TAZ axis, initiated by ECM stiffness (Figure 2), is recognized as one of the fundamental mechanotransduction mechanisms shaping tumor progression [43] and is considered one of the main reasons why 3D culture systems reflect tumor biology more realistically than 2D models.

#### 1.2.2. Spheroids

Sutherland and colleagues, building on the limitations of 2D cell culture methods, developed three-dimensional multicellular tumor spheroids that better mimic in vivo tissues [44]. Spheroids are three-dimensional cell aggregates formed from cancer cell lines or primary cells under non-adherent culture conditions (Figure 1). These structures more realistically replicate the gradients of nutrients, oxygen, and pH observed in solid tumors [45]. The outer layer of spheroids includes proliferative cells, the middle region contains quiescent cells, and the core is typically necrotic [46]. These structural features make spheroids a more physiologically relevant and representative model than two-dimensional monolayer cultures for studying processes such as tumor growth, cellular heterogeneity, and treatment resistance.

Although spheroid models better represent the three-dimensional structure of tumors, they initially lacked the biochemical and mechanical signals provided by the ECM [47,48]. In 1982, Mina Bissell and her colleagues showed that the ECM is not merely a passive scaffold that supports cells but rather an active regulator that directly influences gene expression and cellular functions by organizing tissue architecture [48]. The behavior of cells in culture is strongly influenced by the microenvironment composed of surrounding proteins and signaling molecules [47,49]. This signaling network regulates key mechanisms that control cell differentiation, proliferation, and tumor progression.

In the 1980s, the development of Matrigel, a gel-like protein mixture that mimics the basement membrane, provided experimental support for the active role of the ECM [50]. Matrigel and reconstituted basement membrane (rBM) systems enabled cells to be studied under more physiological conditions in a three-dimensional environment by providing both structural support and essential biochemical signals [51]. In this way, they clearly demonstrated the critical impact of the tumor microenvironment in cancer development.

##### Molecular Characteristics of Spheroids

Spheroids represent important models that reflect the 3D organization and microenvironmental heterogeneity of solid tumors and more realistically reproduce in vivo tumor biology, particularly through the formation of hypoxic regions resulting from restricted oxygen and nutrient diffusion [52,53]. As spheroid diameter increases, oxygen availability decreases within the central regions, leading to the stabilization of hypoxia-inducible factor-1 alpha (HIF-1α) [52,54]. Stabilized HIF-1α translocates to the nucleus and activates transcriptional programs that regulate the expression of numerous target genes [55].

In this context, increased expression of vascular endothelial growth factor (VEGF) promotes angiogenic signaling, whereas upregulation of the glucose transporter GLUT1 enhances glucose uptake and facilitates metabolic adaptation to hypoxic conditions [54,55]. These molecular alterations contribute to tumor cell survival under oxygen-deprived conditions while simultaneously supporting the establishment of a microenvironment that promotes tumor progression [52,55]. Hypoxia-driven molecular reprogramming also plays a central role in the development of treatment resistance [54,55]. Activation of HIF-1α increases the expression of MDR1 and its encoded efflux transporter P-glycoprotein (P-gp), a member of the ABC transporter family, thereby facilitating the active export of chemotherapeutic agents from tumor cells [52,55].

Consequently, drug penetration and intracellular drug accumulation are reduced in spheroids, resulting in levels of chemoresistance that are rarely observed in conventional 2D cultures [53]. Furthermore, the physiological barriers created by the hypoxic core and the intrinsic cellular heterogeneity of spheroids further limit therapeutic efficacy, enabling these models to faithfully reproduce clinically relevant resistance mechanisms [52,53]. Therefore, the HIF-1α–VEGF axis together with MDR1/P-gp-mediated drug efflux pathways are considered key molecular determinants underlying the ability of spheroids to mimic tumor microenvironmental conditions and therapeutic responses [55].

#### 1.2.3. Organoids

Although organoids are not actual human organs, they exhibit many features that resemble in vivo tissues under in vitro conditions [56,57]. Organoids are generally classified into two main groups: tissue-derived organoids and pluripotent stem cell-derived organoids [58]. Patient-derived tumor organoids (PDTOs) are developed by culturing tissues obtained through biopsy or surgical procedures in a three-dimensional matrix environment [56]. Therefore, they largely preserve the genetic and phenotypic features of the tumor tissue from which they originate.

Successful organoid models have been developed from many tumor types, including colon [59], prostate [60], stomach [61], brain [62], and breast cancers [63]. These approaches allow the study of tumor development mechanisms and mutations in signaling pathways. At the same time, they provide significant potential for anticancer drug screening and the development of personalized treatment strategies [56,57,58].

To enhance the biological relevance of organoid models, efforts to reconstruct the tumor immune microenvironment (IME) by incorporating immune cells into the system have gained momentum [64]. In addition, the transplantation of PDTOs into in vivo environments [65] and their integration with microfluidic tumor-on-a-chip systems [35] contribute to more realistic modeling of tissue–tumor interactions and circulation dynamics. These technologies not only support the long-term viability of organoids but also enable dynamic investigation of angiogenesis, drug responses, and cellular interactions [66]. On the other hand, alternative culture systems, such as the air–liquid interface (ALI), provide advantages, particularly in modeling tissues, like the respiratory epithelium, and allow the preservation of more complex cellular components [67].

### 1.3. Preservation of Tumor Genomics and Signaling Pathways in Organoids

PDOs are regarded as the gold standard models in cancer research because they can preserve the genetic and molecular characteristics of the tumor tissue from which they originate with a high degree of accuracy [68]. Unlike traditional cell lines, PDOs can maintain driver mutations and associated signaling pathways that play a critical role in tumor development, even under long-term culture conditions [69]. It has been demonstrated that organoids in key driver genes, such as APC, TP53, and KRAS, are preserved in colorectal and lung cancer organoids; similarly, it has been reported that molecular alterations associated with EGFR and PIK3CA are also stably maintained in organoid models [70]. Transcriptomic and genomic analyses reveal that PDOs maintain the activity of signaling pathways of central importance in tumor biology, such as Wnt/β-catenin, RAS/MAPK, and PI3K/Akt [71].

Furthermore, their high degree of similarity to primary tumors in terms of copy number alterations (CNAs), somatic mutations, and gene expression profiles (median 11.67% genomic discordance) supports the notion that organoids can preserve the biological integrity of the tumor genome [72]. One of the key advantages of PDOs is their ability to reflect both inter-patient and intratumor heterogeneity [73]. By preserving the different cellular subpopulations and molecular subclones found in parental tumors, organoids enable the investigation of processes such as tumor evolution, clonal selection, and the development of treatment resistance [74].

Thanks to these characteristics, organoids are used as powerful platforms not only in the study of tumor biology but also in precision medicine applications [75]. In recent prospective studies, it has been reported that ex vivo drug responses observed in organoids show a high degree of correlation with patients’ clinical treatment outcomes (PPV: 0.78, NPV: 0.80) and can predict both progression-free survival (PFS) and overall survival (OS) durations [76]. The combined use of genomic profiling with high-throughput drug screening approaches will further enhance the role of organoid-based models in optimizing patient-specific treatment selection in the future [77].

## 2. Cellular Responses to Anticancer Drug Treatments

In cancer research, cell culture is extensively utilized in new drug discovery and the development of treatment strategies [78,79]. In 2D systems, deviations from the natural morphology of cells, loss of differentiation capacity, limited cell–cell communication, and artificial uniform exposure to nutrients and drugs restrict the ability to produce treatment responses like in vivo conditions [9,10]. This situation may also contribute to higher rates of cell proliferation. In this context, multicellular tumor spheroids (MCTSs) are widely used to investigate tumor responses to various treatments, including chemotherapy, targeted therapies, and drug delivery systems [80].

Fontoura and colleagues conducted a study to compare the effects of 2D and 3D cell culture models on cell behavior. In the study, two different cell lines obtained from mice, B16F10 melanoma and 4T1 breast cancer, were treated with the chemotherapeutic agents Dacarbazine and Cisplatin. In all 3D models, cells formed spheroids or tissue-like aggregates, and greater cell–cell and cell–ECM interactions were observed compared to 2D cultures. In drug response analyses, cells in 3D cultures were found to be significantly more resistant to chemotherapeutic agents. This finding highlights that 3D cultures better reflect the resistance observed in clinical settings [79]. Studies conducted on different cancer types have similarly shown that drug responses in 3D spheroids are more realistic. In another study, Smabers and colleagues aimed to compare the drug responses of PDTOs with the clinical responses of patients by generating organoids from tissue samples obtained from 23 metastatic colorectal cancer patients. The study found a moderate relationship between the drug responses of the organoids and the patients’ responses to treatment. These findings suggest that organoids can be used as biomarkers in personalized treatment planning and provide results consistent with clinical responses [81]. Similarly, in another study, PDTOs derived from patients with advanced-stage ovarian cancer were used to evaluate responses to both conventional chemotherapy and PARP inhibitors. The response to chemotherapy was found to be highly consistent with the patients’ clinical responses. The study demonstrates that PDTOs can be a powerful tool for predicting responses to chemotherapy and PARP inhibitors in ovarian cancer and may contribute to personalized treatment planning [70].

### Molecular Mechanisms of Drug Resistance in 3D Cultures

**Figure 3:**
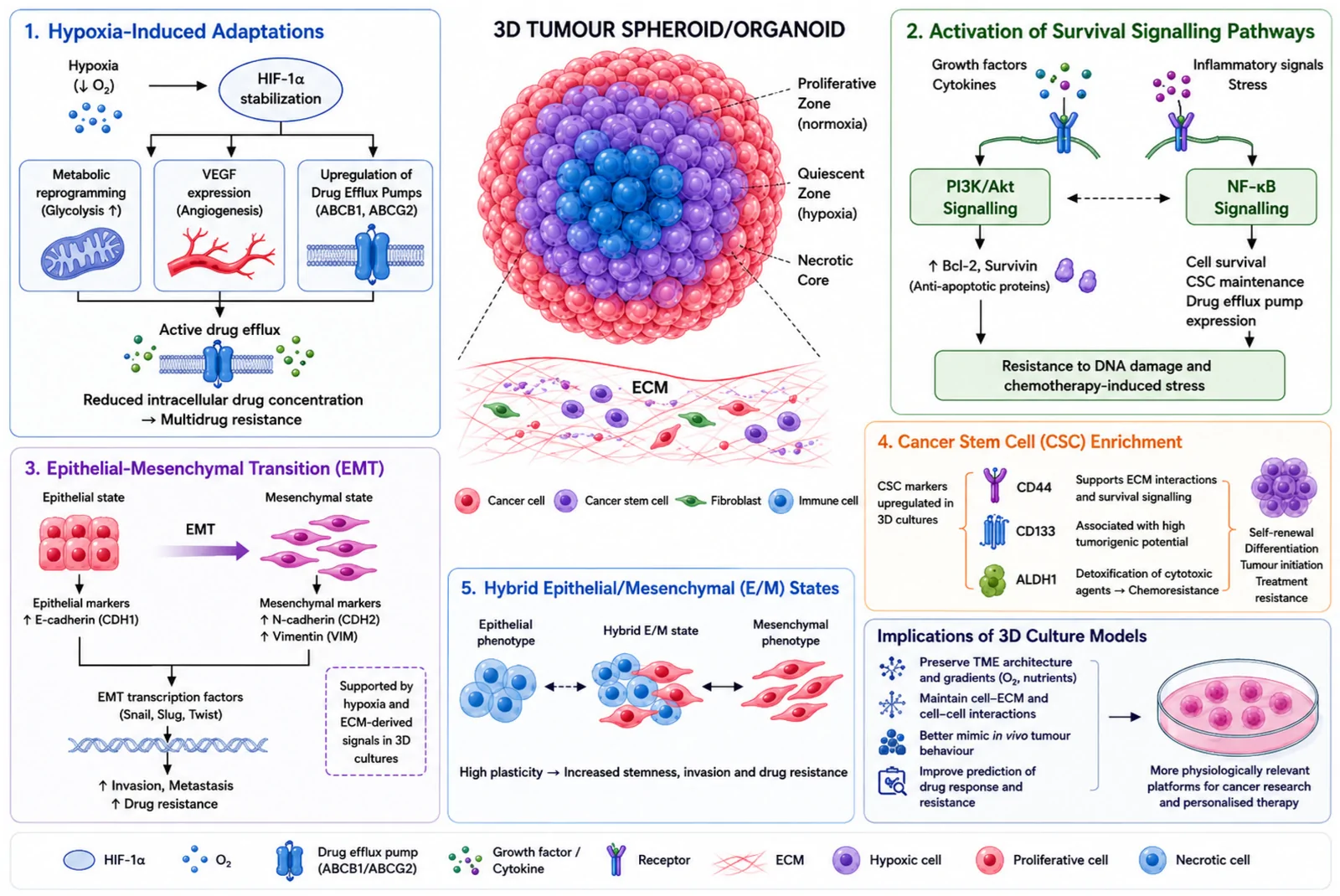
Overview of the molecular mechanisms driving drug resistance in three-dimensional (3D) tumor models. This figure was generated using ChatGPT (OpenAI; GPT-5.6 Luna) through iterative, content-specific prompts specifying the major molecular and cellular mechanisms contributing to therapeutic resistance in 3D tumor models, including hypoxia-induced adaptation and HIF-1α stabilization, upregulation of drug efflux pumps (ABCB1, ABCG2), activation of survival signaling pathways (PI3K/Akt and NF-κB), epithelial–mesenchymal transition (EMT), cancer stem cell (CSC) enrichment (CD44, CD133, ALDH1), plastic hybrid epithelial/mesenchymal (E/M) states, and the physiological relevance of 3D microenvironmental gradients. The generated output was subsequently reviewed and edited by the authors to improve its scientific accuracy, mechanistic detail, visual clarity, resolution, and consistency with the literature. The authors take full responsibility for the scientific content, interpretation, and final version of the figure.

Drug resistance observed in 3D culture systems is driven by multi-layered molecular mechanisms that develop under the influence of the biochemical and physical conditions created by the tumor microenvironment [82,83]. At the heart of this complex ecosystem lies the dynamic interaction between ECM stiffness and hypoxia, which collectively determines cancer progression and response to treatment (Figure 3) [84].

As cancer progresses, excessive activation of cancer-associated fibroblasts (CAFs) and enhanced collagen cross-linking by lysyl oxidase (LOX) increase ECM stiffness [85]. This mechanical change is detected by mechanosensors, such as integrins and Piezo1, on the cell membrane, activating signaling pathways including FAK/Src, Rho/ROCK, and YAP/TAZ [86]. Upon activation, YAP/TAZ translocates to the nucleus to initiate the transcription of genes that support cell proliferation, survival, and drug resistance [85]. Concurrently, this densified and stiffened matrix creates a physical barrier impeding oxygen diffusion, thereby triggering a state of localized hypoxia within the tumor focus [87]. Hypoxia-inducible factors (HIF-1α and HIF-2α), which are stabilized under hypoxic conditions, create a “vicious cycle” by further increasing the expression of matrix metalloproteinases (MMPs) and LOX, leading to further stiffening of the matrix [88,89]. Beyond structural remodeling, hypoxia alters metabolic programming by upregulating glycolytic enzymes (GLUT1, PKM2) and supports VEGF-mediated angiogenic signaling [90,91,92]. Crucially, hypoxia and ECM stiffness synergistically drive multidrug resistance through the upregulation of ABC transporters. HIF-1 directly binds to the ABCG2 promoter region via HIF-1 and increases the expression of drug efflux pumps, such as ABCB1 (MDR1) and ABCG2 [90,93]. In parallel, matrix stiffness activates these transporters (particularly ABCB1/P-gp, ABCC1, and ABCG2) via the integrin/FAK/ERK pathway, conferring resistance to drugs such as doxorubicin and cisplatin [43,94]. These transporters facilitate the active efflux of chemotherapeutic agents from the cell, thereby reducing intracellular drug concentrations and rendering treatment ineffective [83].

In addition to hypoxia-induced adaptations, treatment resistance in 3D cultures is further reinforced by the sustained activation of signaling pathways regulating cell survival [95,96]. Growth factors and cytokines present in the TME activate the PI3K/Akt signaling pathway, thereby increasing the expression of proteins such as Bcl-2 and survivin that suppress apoptosis [95]. Furthermore, NF-κB signaling, which is closely associated with the PI3K/Akt axis, plays a key role not only in regulating inflammatory responses but also in cell survival, the maintenance of the CSC phenotype, and the control of the expression of drug efflux pumps [96,97]. The reciprocal interaction between these pathways causes tumor cells to become more resistant to chemotherapy-induced DNA damage and cellular stress, ensuring that treatment responses observed in 3D culture models more closely resemble in vivo tumor behavior [83].

Three-dimensional tumor models provide suitable platforms for investigating molecular changes associated with EMT due to their ability to preserve the structural and biochemical properties of the TME [98,99]. Both hypoxia and ECM stiffness are key factors that induce the EMT process, whereby epithelial cells lose their polarity and acquire invasive properties [87]. Whilst HIF-1 directly activates transcription factors, such as SNAIL, TWIST, and ZEB1, matrix stiffness supports this process via YAP/TAZ and TGF-β signaling [87]. During EMT, epithelial cells lose cell–cell junctions and polarity, thereby acquiring a more motile and invasive phenotype [100]. Consequently, the expression of the epithelial marker E-cadherin (CDH1) decreases, whilst levels of N-cadherin (CDH2) and Vimentin (VIM) increase [101]. Transcription factors such as Snail, Slug, and Twist enhance the invasion and metastasis capacity of tumor cells by suppressing epithelial genes and activating mesenchymal gene programs [98,101].

Another feature closely associated with EMT is the enrichment of CSC phenotypes [100]. Hypoxic TME and stiffened matrix enhance the self-renewal capacity of CSCs and tumor heterogeneity by upregulating pluripotency factors, such as OCT4, SOX2, and NANOG, as well as CSC markers, such as CD44, CD133, and ALDH1 [89,102]. CD44 supports interactions with the extracellular matrix and survival signals, whilst CD133 is associated with high tumor-forming capacity, and ALDH1 contributes to chemotherapy resistance by playing a role in the detoxification of cytotoxic agents [103]. Recent studies indicate that tumor cells often exist in hybrid epithelial/mesenchymal (E/M) states, exhibiting both characteristics simultaneously [100,101]. These highly plastic cell populations are associated with increased stem cell properties, invasive capacity, and treatment resistance, and 3D culture systems offer significant advantages in investigating these dynamic processes [99,104].

## 3. Conclusions

The 3D cell culture systems have been widely adopted in biomedical research in recent years, as they address many of the limitations of traditional 2D models. Compared to 2D models, 3D systems provide a more realistic environment for cell culture; therefore, their responses to drug treatments are also more representative. Organoids and PDTOs allow cells to grow under conditions closer to their natural environment, reflecting the structure of tumors and the interactions between different types of cells better. This contributes to a more accurate evaluation of drug effects on cells and supports the development of personalized treatment approaches. Properly mimicking the ECM, one of the key factors determining cell behavior, also plays a critical role in the success of these systems. For this purpose, technologies such as hydrogels, microfluidic systems, and air–liquid interface platforms have been developed.

### 3.1. Technical Limitations, Standardization, and Reproducibility Issues

Although 3D culture models, particularly patient-derived tumor organoids and spheroids, provide significant advantages in cancer research, they also have several biological and technical limitations. One of the main issues is the lack of standardization. Differences in culture conditions, media composition, ECM composition, cell density, culture duration, and laboratory practices can lead to variability in results. Variations in drug responses even among organoids derived from the same tumor indicate that intratumoral heterogeneity is reflected in the model and may limit its predictive power in clinical settings [37]. In addition, many models rely on rBM, such as Matrigel, derived from mouse tumors, and its batch-to-batch variability in protein composition and mechanical properties can substantially affect cell growth, differentiation, morphology, and drug responses, thereby reducing experimental standardization and reproducibility [5,105]. The inability of rBM to fully replicate the fibrillar architecture, biochemical composition, and mechanical properties of natural tumor ECM can also lead to incomplete representation of important biological processes, such as cell migration and invasion. Synthetic and chemically defined hydrogels have, therefore, emerged as potential alternatives because they provide tunable mechanical properties and more precisely controlled stiffness, degradability, ligand presentation, and other physicochemical features, potentially improving reproducibility and enabling a more consistent and precise recreation of the tumor microenvironment. Furthermore, most organoid models lack sufficient vascular structures and do not fully include stromal and immune components of the tumor microenvironment [106]. Although co-culture approaches attempt to address these limitations, factors such as cell sourcing, cost, and technical complexity remain significant challenges.

Similarly, spheroid models also present important biological limitations. Cancer cell lines do not represent the entire patient population, as each patient has a unique molecular profile [107]. Therefore, cell line-based spheroids may not adequately reflect patient-specific tumor heterogeneity. In addition, cell lines may accumulate genetic and epigenetic changes during long-term culture. These changes can cause cells to diverge from their original tumor characteristics, potentially affecting the reliability of experimental results obtained from spheroid models [108]. Another major limitation of 3D spheroid models is the restricted diffusion of oxygen, nutrients, and therapeutic agents within larger cultures. As spheroids increase in size, particularly beyond approximately 200–400 µm, they encounter an oxygen diffusion limit in which oxygen and nutrient transport become insufficient to meet the metabolic demands of cells located in the inner regions. This can result in steep gradients of oxygen and nutrients, leading to hypoxia, nutrient deprivation, and the formation of a necrotic core. Although these gradients can reproduce certain features of solid tumors, excessive or uncontrolled core necrosis may introduce experimental variability and complicate the interpretation of drug–response studies. Similarly, limited penetration of therapeutic compounds into the spheroid core can result in apparent drug resistance that is partly attributable to physical diffusion barriers rather than solely to cellular resistance mechanisms. Static culture conditions can further exacerbate these limitations because they lack the dynamic fluid flow required to support continuous gas and nutrient supply and efficient waste removal.

Beyond these biological limitations, practical and logistical constraints also affect 3D culture systems. The establishment and maintenance of organoids and spheroids require longer culture periods compared to 2D monolayers, which increases labor intensity and demands continuous monitoring. The materials used, including specialized hydrogels, growth factors, and microfluidic devices, are often expensive, limiting scalability for large-scale drug screening. Additionally, some 3D models are more susceptible to contamination, necessitating stringent aseptic techniques and advanced laboratory infrastructure [109]. Another important consideration is the need for assay normalization. Differences in spheroid or organoid size, cell number, viability, culture duration, drug exposure conditions, and endpoint measurements can influence experimental outcomes and make comparisons between studies difficult. Therefore, the use of defined and consistently reported experimental parameters, together with standardized protocols for culture establishment, drug treatment, viability assessment, imaging, and quantitative analysis, is essential for improving inter-laboratory reproducibility and facilitating reliable comparisons of 3D culture data.

Emerging technologies such as tumor-on-a-chip and other micro-physiological systems may help overcome several of these limitations by integrating microfluidic systems with controlled fluid flow, vascular-like perfusion, oxygen and nutrient gradients, and dynamic cell–matrix interactions. By mimicking vascular perfusion and mechanical forces such as fluid shear stress, tumor-on-a-chip technologies can facilitate gas and nutrient delivery and waste removal, thereby helping to overcome diffusion constraints and providing more physiologically relevant, miniaturized environments for drug testing and disease modeling. Together with synthetic hydrogels, these approaches offer promising strategies for reducing matrix-related variability, improving control over tumor architecture and mechanical properties, and more accurately reproducing the complex spatial and physiological conditions of human tumors.

Nevertheless, improved standardization and reproducibility alone will not fully resolve the challenges associated with clinical translation. Although patient-derived organoids and spheroids can preserve certain molecular and phenotypic characteristics of the original tumor, they may not fully reproduce the vascular, stromal, immune, and systemic components that influence therapeutic responses in patients. Consequently, drug responses observed in 3D cultures may not always directly correspond to clinical outcomes. Inter-patient variability, differences in tumor evolution, pharmacokinetic and pharmacodynamic factors, and the absence of a complete tumor microenvironment can further limit the predictive value of these models. Integrating patient-derived 3D models with standardized assay procedures, synthetic matrices, co-culture systems, and tumor-on-a-chip platforms may, therefore, provide a more reliable approach for bridging the gap between experimental drug testing and clinical application.

### 3.2. Future Perspectives and Emerging Technological Approaches

The future of 3D culture technologies is advancing alongside organ-on-a-chip systems and artificial intelligence-supported analyses. Organ-on-a-chip platforms create more dynamic and physiologically relevant environments through microfluidic systems [110]. This allows for more realistic investigation of drug absorption, distribution, and toxicity processes. Artificial intelligence and machine learning facilitate the analysis of large datasets, improve imaging quality, optimize experimental workflows, and enhance predictive accuracy [111]. In addition, new matrix approaches such as collagen-based ALI systems and hydrogels provide more controlled and physiologically relevant microenvironments as alternatives to rBM [17]. In subsequent studies, these advanced 3D models, integrated with genomic and single-cell analyses, are predicted to play a significant role in the development of personalized treatment strategies. However, achieving this will require improved standardization, reduced costs, and increased clinical validation studies.

Finally, to facilitate comparison of the main experimental platforms currently used in cancer research, Table 1 summarizes their principal characteristics, advantages, limitations, and representative applications. Overall, cancer cell culture models show a progressive increase in biological and translational relevance as their complexity increases. 2D monolayer cultures offer low cost, high throughput, simple imaging, and high reproducibility, but poorly reproduce the three-dimensional architecture, extracellular matrix (ECM) interactions, tumor microenvironment (TME), and heterogeneity of tumors, limiting their predictive value [112,113]. Spheroids better reproduce 3D architecture, cell–cell interactions, and oxygen and nutrient gradients, making them useful for studying drug penetration and resistance, although diffusion limitations and the limited representation of stromal and immune components remain important drawbacks [114]. Patient-derived organoids (PDOs) provide greater tumor heterogeneity and preserve patient-specific genetic and phenotypic characteristics, offering strong potential for personalized therapy, but their dependence on Matrigel, batch variability, and incomplete vascular and immune components limit standardization [115,116]. Organ-on-chip/tumor-on-a-chip systems provide high physiological relevance by incorporating microfluidic perfusion, vascular-like flow, mechanical forces, and real-time monitoring, although their technical complexity, cost, and limited throughput remain challenges [117]. Finally, co-culture systems enable the integration of stromal, immune, and endothelial components, providing a more comprehensive representation of the TME and high potential for translational applications, but they are affected by donor variability, optimization requirements, and difficulties in standardization [118].

## Figures and Tables

**Table 1:** Critical comparison of conventional 2D cultures, spheroids, patient-derived organoids, organ-on-chip systems, and co-culture platforms in cancer research.

Model	Major Advantages	Major Limitations	Tumor Microenvironment Representation	Tumor Heterogeneity	Drug Screening and Response	Standardisation and Reproducibility	Clinical Translation
2D monolayer culture	Simple, low cost, high throughput, easy imaging and quantification	Lacks 3D architecture, ECM interactions, and physiological gradients	Low	Low	High throughput but poor predictive value	High reproducibility	Limited predictive power
Spheroids	3D architecture, cell–cell interactions, hypoxia and nutrient gradients	Diffusion limits (>200–400 µm), necrotic core, limited stromal/immune components	Moderate	Low–moderate	Good for penetration and resistance studies	Moderate	Moderate
Patient-derived organoids (PDOs)	Preserve patient-specific genotype/phenotype, strong translational relevance	Matrigel dependence, batch variability, lack of full vasculature/immune system	Moderate–high	High	Strong predictive potential for personalized therapy	Moderate–low	High potential
Organ-on-chip/Tumor-on-a-Chip	Microfluidic perfusion, vascular-like flow, mechanical forces, real-time monitoring	Technically complex, expensive, low-to-moderate throughput	High	Moderate–high	High physiological relevance in drug testing	Moderate	High potential
Co-culture systems	Includes stromal, immune, endothelial interactions; improves TME modeling	Complex optimization, donor variability, difficult standardization	High	High	Useful for immune and stromal drug response studies	Moderate–low	High potential

Note: ECM, extracellular matrix; PDOs, patient-derived organoids; TME, tumor microenvironment. The qualitative ratings represent an integrated assessment of the relative performance of each model class based on the cited literature.

## Data Availability

Data is contained within the article. During the preparation of this manuscript/study, the authors used NotebookLM exclusively for language editing and grammar correction. Also, ChatGPT (OpenAI; GPT-5.6 Luna) was utilized solely for the preparation of figures. The authors have reviewed and edited all AI-assisted output and take full responsibility for the content of this publication.

## Abbreviations

The following abbreviations are used in this manuscript:

2D	two-dimensional
3D	three-dimensional
ABC	ATP-binding cassette (transporters)
CSC	cancer stem cell(s)
ECM	extracellular matrix
EMT	epithelial–mesenchymal transition
FAK	focal adhesion kinase
IME	immune microenvironment
MCTS	multicellular tumor spheroids
PDTOs	patient-derived tumor organoids
PFS	progression-free survival
rBM	reconstituted basement membrane
TME	tumor microenvironment
VEGF	vascular endothelial growth factor
